# Selective Sentinel Node Dissection in Melanoma with Trends and Future Directions

**DOI:** 10.3390/cancers16213625

**Published:** 2024-10-27

**Authors:** Eric Pletcher, Mark B. Faries

**Affiliations:** The Angeles Clinic and Research Institute, A Cedars-Sinai Affiliate, 11800 Wilshire Blvd., Los Angeles, CA 90025, USA; ericpletcher@gmail.com

**Keywords:** melanoma, sentinel lymph node, prognosis

## Abstract

Starting with its earliest descriptions, melanoma has been recognized as a tumor with a predilection for metastasis to regional lymph nodes. This tendency led to initial recommendations for very aggressive early surgical management of the regional nodal basin. However, those recommendations were the source of much controversy over nearly a century, until the minimally invasive surgical technique of sentinel lymph node (SLN) biopsy was developed by Morton, Cochran and colleagues. This technique has been evaluated in a series of prospective clinical trials, which have clarified its role and the management of lymph nodes in this disease. Current controversies relating to SLN biopsy include optimal selection of patients for the procedure, the role of gene expression profiling in initial melanoma management, and the potential therapeutic effects of SLN biopsy-based management. In addition, the SLN appears to be a rich source of data relating to the host–tumor interface and the immune microenvironment, which may advance our understanding of the biology of melanoma. Finally, although the surgical technique is well developed at this point, there may be additional technical improvements that are possible as well.

## Simple Summary

The most frequent initial site of metastasis for patients with melanoma is the regional lymph node basin. Management of regional lymph nodes was transformed through the development of lymphatic mapping and sentinel lymph node biopsy. Through a series of prospective multicenter clinical trials, the role of sentinel lymph node biopsy has been established and clarified. Although areas of controversy remain, the procedure now has a well-defined role in treatment and holds promise as a source of valuable information to guide our understanding of melanoma biology and treatment into the future.

## 1. Introduction

### History and Rationale for the Importance of Lymph Nodes in Melanoma

The Scottish surgeon John Hunter is credited with describing the first case of melanoma in the English literature in 1787 [1]. The specimen associated with this case is still preserved in the Hunterian Museum in London and appears to be a cervical lymph node metastasis rather than a primary skin tumor, which confirms the central importance of regional nodal metastases in this disease even from its earliest history. The treatment for the disease was quite limited in the following years, but as medical science advanced and surgery improved, newer treatment options were developed. One significant actor in this development was Herbert Snow, a London surgeon [2]. In 1892, Snow published a treatise on melanoma treatment in *The Lancet* [3]. In his paper, he described several cases and recommended treatments. One particular focus was on the surgical treatment of regional lymph nodes. He noted that there was frequently extension of disease to these nodes that was subclinical at the time of initial diagnosis of the primary skin tumor. He felt that if these regional nodes were left untreated, the disease they contained would grow and progress and that by the time they were detected clinically, it would be too late to save the patient. He therefore recommended “anticipatory gland excision” or surgical removal of regional nodes at the time of diagnosis even if they appeared to be normal on exam.

Over the next century, anticipatory gland excision became known as elective lymph node dissection (ELND) and was the subject of much controversy. On one hand, it was clear that the pattern of metastasis in many cases of melanoma was initially through lymph nodes, suggesting the potential value of early removal of those sites, but on the other hand, this surgery was accompanied by a significant risk for short—and long-term—morbidity, and many of the patients who underwent the procedure had no melanoma found in the nodes after removal. Eventually, this led to multiple randomized clinical trials to evaluate the utility of ELND. These included trials at the Mayo Clinic, as well as trials through the World Health Organization and led by investigators in Italy [4,5,6]. These trials did not show a significant survival benefit associated with ELND but were hampered by sample size limitations and an inability to accurately stratify patients at the time or randomization. The last ELND trial was conducted by the Intergroup and was led by Charles Balch [7]. This trial, and some of the prior ELND trials, showed a trend toward improved survival with ELND, but this did not reach statistical significance. In the Intergroup trial, several subgroup analyses, which had been pre-specified, did show significant benefits, including their intermediate thickness (1–2 mm primary tumor thickness) group, those with non-ulcerated primary melanomas, patients younger than 60 and those with extremity melanomas. Therefore, there was enough evidence to support efficacy to maintain the belief of some clinicians in the value of ELND, but enough evidence of lack of significant efficacy to maintain strong opposition from others.

The introduction of SLN biopsy for melanoma upended the entire discussion and transformed surgical management. The basis for this technique is from the understanding that lymphatic drainage from a primary tumor site is not directed generally toward an entire nodal basin but rather to a single or small number of specific lymph nodes within that basin. That concept had been proposed several previous times, but not in a way that led to an accurate surgical technique. Both Gould and Cabanas described “sentinel” lymph nodes in parotid and penile tumors, respectively [8,9]. They described anatomic locations of lymph nodes whose pathologic status should accurately reflect the overall status of the nodal basin. However, the absence of a practical technique to intraoperatively identify that sentinel node limited the adoption of the concept and meant it could not be applied to a tumor such as melanoma with a variable primary tumor location.

The technique we now recognize as SLN biopsy had its origins in ELND for melanoma. For patients with melanomas in locations with ambiguous lymphatic drainage, it was often unclear which nodal basin should be electively dissected. Morton and colleagues at the University of California Los Angeles applied lymphoscintigraphy to rationally plan ELNDs [10]. In the early studies, they used colloidal gold injected at the primary tumor site to determine which of the potential draining basins were at risk for metastasis. This proved to be an accurate way to plan surgery. As imaging technologies improved, it became apparent that the regional lymphatic drainage was not directed to the entire nodal basin, but rather to a single or small number of nodes within that basin. They hypothesized that the pathologic status of that initial site of drainage would reflect the status of the nodal basin overall and proposed the removal of only that draining SLN node as the initial step in evaluation. If the SLN was clear, the remainder of the nodes in the basin could be left in place.

The initial technique involved lymphoscintigraphy to identify the draining basin and intraoperative injection of vital blue dye. The draining channel was traced to the SLN, which was removed. In the initial studies, this was followed by complete dissection so that the accuracy of the technique could be examined. The first report was presented at the Society of Surgical Oncology in 1990. There was some skepticism about the results and the publication was delayed until 1992 [11]. However, this manuscript has become one of the most highly cited papers in surgical oncology in the years since then. In this series, the SLN was found in 82% of nodal basins and contained metastases in 21% of cases. Non-SLNs were the exclusive site of metastases in only 2 of 194 dissections, with a total of 3079 lymph nodes. The technique was subsequently improved by the addition of intraoperative use of a gamma probe to detect the radioactivity of the tracer injected for the lymphoscintigram, yielding a success rate for the procedure that is now closer to 100% [12].

## 2. Clinical Trials

### 2.1. MSLT-I

**Figure 1:**
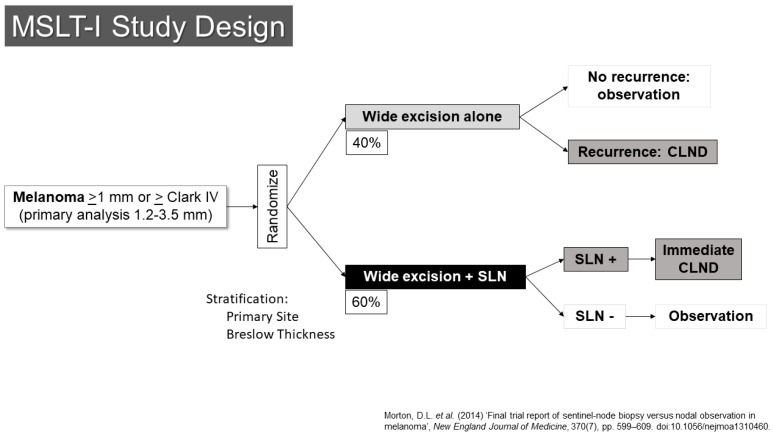
Study schema for MSLT-I [14].

The SLN concept was quickly moved to evaluation through prospective, randomized clinical trials. The first Multicenter Selective Lymphadenectomy Trial (MSLT-I) opened to accrual in 1994 (Figure 1) [13]. This trial randomized patients with primary melanomas of at least 1.2 mm in thickness or Clark’s level IV or higher in depth to either wide excision alone (40%) or to wide excision with SLN biopsy (60%). Patients with metastases in their SLNs underwent completion lymph node dissection right away. Those who had wide excision alone would have nodal dissection if they developed regional nodal recurrences. Based on earlier data from the elective lymph node dissection trials and an analysis of institutional databases, it was suggested that the group most likely to have a detectable, statistically significant survival benefit were those patients with melanomas between 1.2 and 3.5 mm in thickness, and those patients were the primary target stratum for the trial. The primary endpoint was melanoma-specific survival.

The trial revealed a number of important findings [14]. First, it confirmed the prognostic significance of the pathologic status of the SLN. Those with SLN metastases had a risk of death that was increased by two and a half times compared to patients with tumor-negative SLNs. There appeared to be progression within the nodal basin of melanoma, as patients whose nodal basin was dissected after an SLN metastasis had an average of 1.4 involved nodes compared to 3.3 positive nodes in the observation group, who underwent dissection at the time of clinical recurrence.

In 2014, results with a 10-year follow-up were published [14]. These did not demonstrate a statistically significant improvement in melanoma-specific survival for patients in the SLN arm of the trial (81% vs. 78%, HR 0.84, 95% Confidence Interval 0.64–1.09, *p* = 0.18). The procedure was associated with a significant improvement in disease-free survival (71% vs. 65%, HR 0.76, 95% CI 0.62–0.94, *p* = 0.01). The prognostic value of the SLN status was also confirmed with greater follow-up (HR for recurrence was 2.64 (95% CI 1.92–3.64), HR for melanoma-specific death 2.40 (95% CI 1.61–3.56)). Although complete node dissection, which was performed for all patients with SLN metastases, was associated with a risk of lymphedema, this risk appeared to be significantly less than the risk that accompanied therapeutic lymph node dissection, which was performed for patients who experienced nodal recurrence in the observation arm (12.4% vs. 20.4%, *p* = 0.04) [15]. With adequate follow-up time, the proportion of patients who developed nodal recurrence became virtually identical with the number of those who had nodal disease in the SLN arm of the trial (at 10-years, 21.9% in SLN vs. 19.5% in observation for intermediate thickness, 42.0% in SLN vs. 41.4% in thick primaries).

### 2.2. Sunbelt

**Figure 2:**
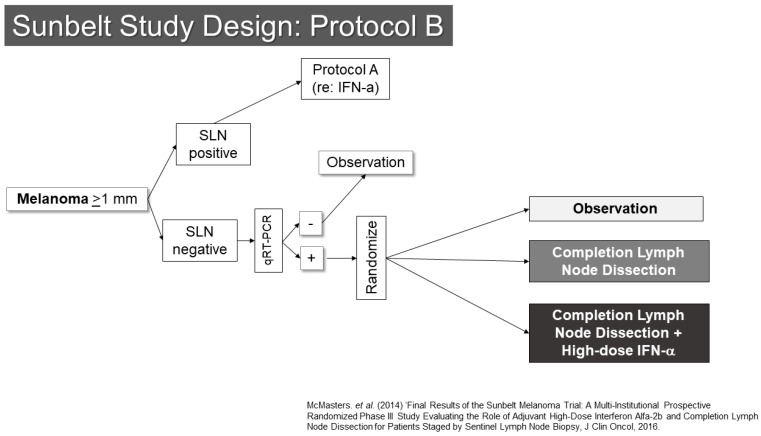
Study Schema: Sunbelt Melanoma Trial [18].

Another trial involving SLN biopsy was the Sunbelt Melanoma Trial (Figure 2) [16,17,18]. This study enrolled patients with melanomas at least 1 mm in thickness. The trial examined the value of high-dose interferon-alpha in one part (Protocol A) and the utility of complete node dissection for patients whose SLNs were negative by standard pathology, but positive for melanoma by an RT-PCR assay (Protocol B). Protocol A did not show a benefit to interferon. Protocol B showed an interesting trend toward improved outcomes for patients with RT-PCR positive SLN who underwent completion dissection (*p* = 0.063). As noted below, the second Multicenter Selective Lymphadenectomy Trial results did not show similar findings for completion lymph node dissection or RT-PCR evaluation of SLNs. From the Sunbelt Trial, the “10% rule” also gained prominence. This rule suggested that if all blue stained SLN and all nodes with at least 10% of the counts of the hottest SLN were removed, the false-negative rate would be expected to be very low (<1%). This appears to be a reasonable rule of thumb, though there is generally a substantial difference between the radioactivity of a node when assessed in vivo compared to when it has been removed. This makes clinical judgement an important aspect of determining when enough SLNs have been removed. Some additional useful information may be garnered from lymphoscintigraphy, provided that that the imaging is of sufficient quality to determine whether drainage to multiple nodes is in a parallel or serial fashion.

As experience with SLN biopsy grew, it became apparent that in most cases, the larger, completion lymph node dissection yielded no additional nodal disease [19]. That is, all of the nodal disease had been removed through the SLN biopsy, and the larger, potentially more morbid nodal dissection did not lead to additional disease removal. This led to the clinical question of whether the larger procedure was necessary, and two clinical trials were conducted to answer that question.

### 2.3. DeCOG-SLT and MSLT-II

**Figure 3:**
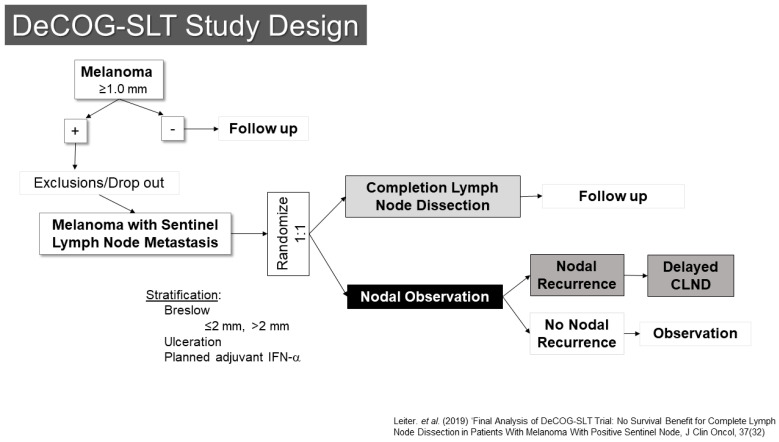
Study Schema: DeCOG-SLT [21].

One of these trials, conducted by the Dermatologic Oncology Group (DeCOG-SLT), enrolled patients at 41 German melanoma centers (Figure 3) [20,21]. Between 2006 and 2014, 5547 patients were screened, of whom 1269 had SLN metastases. Of those, 483 ended up being randomized (1:1) to either completion lymph node dissection or to observation. The final results were reported in 2019, showing no difference in the primary endpoint of distant metastasis free survival (HR CLND vs. observation 1.08 90% CI 0.83–1.39, *p* = 0.65). Interestingly, there was also no difference in recurrence free survival, with only a modest reduction in regional nodal recurrence (10.8% vs. 16.3%) for patients undergoing dissection, although the trial had somewhat limited statistical power to detect differences.

**Figure 4:**
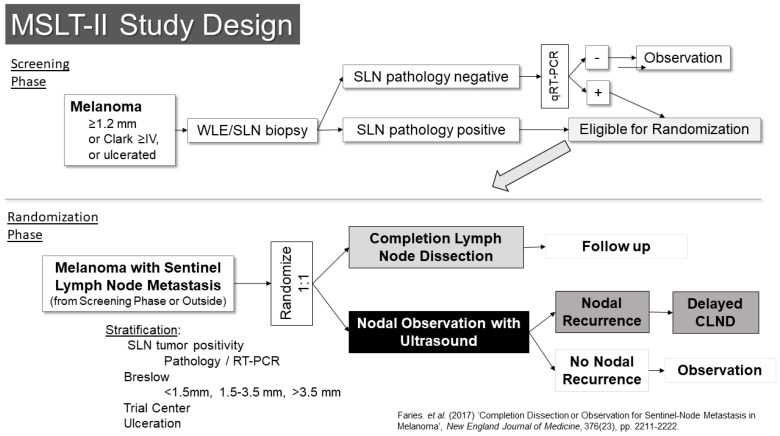
Study Schema: MSLT-II [22].

The other trial examining this question was the second Multicenter Selective Lymphadenectomy Trial (MSLT-II) (Figure 4) [22]. This trial included a screening phase, in which patients were enrolled prior to SLN biopsy. Eligibility for the screening phase was restricted to patients with melanomas of at least 1.2 mm in thickness, Clark’s level IV or ulcerated melanoma. Patients who were in the screening phase underwent an ultrasound of the SLN basin prior to surgery to evaluate the utility of ultrasound screening. In that setting, the ultrasound was not found to be particularly useful, with a sensitivity of only 7.1% for detecting metastases identified in subsequent SLN biopsies [23]. The other main activity in the screening phase was RT-PCR evaluation of SLNs that were free of pathologically evident metastases using standard pathology assessments. Prior retrospective analysis suggested these patients might have a similar risk for recurrence as pathology-positive patients, albeit over a delayed time course. Follow-up of these patients in the trial, however, suggests their outcomes are better than what had been expected. Patients whose SLNs were positive by RT-PCR were then eligible for randomization in the second phase of the trial.

The randomization phase of the trial was open to patients who had SLN metastases (by pathology or RT-PCR) from the screening phase as well as patients who were found to have pathologic metastases outside the screening phase. These patients (n = 1934) were randomized 1:1 to either completion lymph node dissection or to nodal observation using nodal ultrasound in addition to standard follow-up. The results were reported in 2017 and demonstrated no benefit in melanoma-specific survival for patients who underwent completion dissection [22]. There was prognostic information gained from the pathologic evaluation of the remainder of the lymph nodes in the basin, which was independent of other variables (HR for non-SLN metastasis 1.78 95% CI 1.19–2.67, *p* = 0.005) and they had a small but significantly improved recurrence-free survival (68% vs. 63%, *p* = 0.05), which appeared to be based entirely on a reduction in nodal recurrence (HR 0.31, 95% CI 0.24–0.41, *p* < 0.001). Crucially though, there was no indication in an improvement in melanoma-specific survival (HR 1.08, 95% CI 0.88–1.34, *p* = 0.42). With the publication of the results of these two trials, nodal observation became a standard option for patients with SLN metastases, and it is now the preferred option in the National Comprehensive Cancer Network melanoma guidelines.

For nodal observation patients who recur, those with node-only recurrences may undergo complete therapeutic dissection at that point. However, with the now convincing evidence for neoadjuvant treatment for such patients, the extent of surgery after pre-operative immunotherapy remains a subject of some uncertainty. The PRADO trial investigated whether less extensive resection, termed “index node” management, would provide equal outcomes for those patients who experienced a complete or major pathologic response [24]. This trial was negative by the pre-trial criteria, but certainly did not rule out the possibility of reasonable surgical de-escalation for these patients. An “MSLT-III” trial is now in the planning stages to definitively evaluate the question.

## 3. Controversies and Future Directions

### 3.1. Selection for SLN Biopsy

Many patients with melanoma do not require surgical staging of their regional lymph nodes. In general, the selection of patients for the SLN procedure is based on the risk of nodal metastasis. There are several methods for making this estimate. The simplest method is using the clinical American Joint Commission on Cancer (AJCC) staging system [25]. In this system, melanomas <0.8 mm without ulceration are cT1a. Those with melanomas 0.8–1 mm in thickness or those with thinner melanomas with ulceration are T1b. Those with melanomas >1 mm in thickness are at least T2a. Regarding SLN biopsy, the guidelines recommend discussing and offering the procedure for melanomas T2a and above, discussing and considering the procedure for those whose with T1b melanomas and generally not considering it for patients with T1a melanoma unless there are “high-risk” features [26]. There is some uncertainty which features serve as the best indicators of high risk, but they include younger age, lymphovascular invasion, and a high mitotic rate (≥2 mitoses/mm^2^).

While simple, these recommendations may not provide the best estimates of SLN metastasis risk, and several nomograms have been developed to increase precision. These include systems from Memorial Sloan Kettering Cancer Center (https://www.mskcc.org/nomograms/melanoma/sentinel_lymph_node_metastasis), which incorporate factors such as age, Breslow thickness, Clark’s level, ulceration status, and primary tumor location [27]. The Melanoma Institute Australia has a highly validated nomogram (melanomarisk.org.au) that uses age, Breslow thickness, histologic subtype, lymphovascular invasion, ulceration status, and mitotic rate [28]. Both have tools available online to allow free, easy use of the system. One criticism of these nomograms, particularly at the low-risk end of the spectrum is that they are based on populations in which SLN biopsy was performed. That is, they had been selected for SLN biopsy by their treating physicians. This might change the pre-test probability of a positive node. Other prediction schemes have been developed for thin melanomas using patients who have not undergone surgical staging and identifying risk factors for nodal recurrence. One developed at the John Wayne Cancer Institute uses categories for age, Breslow thickness and gender to estimate risk [29]. Finally, there are gene expression profile assays that have been developed with the goal of increasing the accuracy of outcome prediction in melanoma [30]. Some recent analyses using gene expression profiling have demonstrated more accurate risk stratification than that provided by the AJCC staging system, but it is not clear to what extent the estimates provided from these potentially costly tests add to the free information available from existing nomograms [31,32].

Whatever means are used to estimate the risk of SLN metastasis, there is a general consensus that patients with a risk under 5% do not require SLN biopsy, those with risk between 5 and 10% should consider the procedure, and those with risk above 10% should be recommended to undergo it, unless there is a contraindication. The 5% and 10% thresholds recommended in the guidelines might also be taken as just that: guidelines. There are some patients, for example young patients, for whom the value of the information gained from SLN biopsy is of greater value. Treating physicians should be certain to help patients arrive at the best decision for themselves.

One recent controversial area is the use of SLN biopsy among patients with high-risk melanomas who would qualify for adjuvant therapy even if their SLN is free of metastasis. This has arisen with the approval of PD-1 blocking antibodies in the adjuvant setting as a result of the Checkmate 716 and Keynote 76K clinical trials [33,34,35]. These trials demonstrated an improvement in relapse-free survival and distant metastasis free survival, though no demonstrated benefit in overall survival. The argument against SLN biopsy in that setting is that the procedure is no longer required to gain access to the systemic therapy and can be omitted. However, there are several significant concerns with taking that treatment approach. First, though it is unlikely that there is a large survival benefit to SLN biopsy for these thick melanomas, there is a very clearly demonstrated relapse-free survival benefit from the procedure demonstrated in MSLT-I. This difference may be blunted but would not be eliminated by adjuvant systemic therapy. Patients who do suffer nodal recurrence are generally treated with complete nodal dissection, which carries a significantly greater morbidity risk compared to the SLN procedure. It is clear from MSLT-II that most nodal recurrences (approximately 80%) can be avoided by performing the SLN biopsy alone, without the need for complete dissection at any point [33]. Second, although adjuvant systemic therapy is approved in stage IIB and IIC melanoma, it is not clear that every such patient should undergo the therapy. The absence of a demonstrated overall survival benefit and the potential serious or even fatal toxicity of these treatments suggests that a careful discussion about the risks and benefits is required for patients to make an informed decision. Approximately 30–40% of these patients will harbor clinically occult nodal metastases, which would convert them to stage IIIC and decrease their expected 5-year melanoma-specific survival from 87% or 82% to 69%. It seems apparent that this information is important for making an informed choice.

### 3.2. Therapeutic Effects of SLN Biopsy

The therapeutic effect of SLN biopsy is often discounted, which is understandable since MSLT-I did not demonstrate a significant improvement in melanoma-specific survival for those patients undergoing the procedure. However, this may oversimplify the situation in a way that obscures the clinical benefit patients derive. These benefits may come in one of three forms. The first involves more accurate selection of patients for additional therapy and potentially tailoring the choice of adjuvant therapy to the risk they face. As additional combination therapies are examined in this setting, it may be important to balance added toxicity of a medical regimen with a higher risk of recurrence.

More directly on point, though, are two potential therapeutic effects of the procedure itself. As alluded to above, there is a very clear improvement in relapse-free survival with SLN biopsy, particularly with regard to nodal recurrence [14]. It is clear from MSLT-II that this benefit does not require a full lymph node dissection most of the time [36]. The likelihood of durably clearing the nodal basin with SLN biopsy alone can be estimated based on several clinical pathologic factors. These include patient age, primary tumor ulceration, basin site, Breslow thickness, and the tumor burden in the SLNs. Even in high-risk categories, though, most patients do not suffer nodal recurrence. This benefit appears clear and comes at a very modest risk of toxicity, which is almost always short lived.

The question of melanoma-specific survival is certainly more controversial. Often, the negative result of MSLT-I is cited as proof of the absence of a survival impact. However, the trial population demonstrated a lower overall event rate than had been estimated in its statistical design, leaving it somewhat underpowered to adequately address the primary endpoint. It was not designed as an equivalence trial, which would have required a much larger sample size. The fundamental challenge with the trial design was that most patients who are treated have benign SLNs and cannot benefit from their removal. This was examined in MSLT-I through an analysis of the subgroup of patients who had nodal disease [37]. Comparing those whose nodal metastases were detected by SLN biopsy to those that were detected at the time of nodal recurrence, the risk of melanoma-related death was markedly reduced in the SLN group (HR, 0.56 (95% CI, 0.37–0.84) *p* = 0.006) [14]. Since patients were not randomized into the two compared groups, however, there is appropriate concern regarding a comparison of their outcomes. This was addressed with a statistical methodology of latent subgroup analysis that also supported the conclusion that there was a survival advantage for patients with nodal disease. This applied only to the primary aim stratum of the trial (intermediate thickness) and not to the thicker primary subgroup. This pattern of survival benefit in intermediate thickness melanoma and not thicker melanoma had also been seen in earlier elective lymph node dissection trials, suggesting that there may be an underlying biological basis for the observation. Finally, with regard to a survival benefit to SLN biopsy, a population-based approach has been taken to try to overcome the sample size challenge of a clinical trial. A cohort of 1989 patients in the Southeast area of the Netherlands compared patients with intermediate thickness (1.2–3.5 mm) melanomas treated with or without SLN biopsy. This revealed a very similar hazard ratio for benefit in the SLN group (HR = 0.80, 95% CI 0.66–0.96), comparable to that observed in MSLT-I (HR 0.84). However, with the increased sample size, this finding was statistically significant (*p* < 0.001) [38]. This adds to similar findings of improved survival related to SLN biopsy seen in SEER analyses [39,40], though that database may not allow as complete an adjustment for confounding variables. Overall, although there is a lack of level I evidence that SLN biopsy improves melanoma-specific survival, such a benefit does not appear to be ruled out by the existing data. If there is a benefit, it is likely small for the overall population, but it appears to come at a limited cost in terms of long-term morbidity.

### 3.3. SLN as an Investigational Tool

Another aspect of the SLN that is of interest is its potential role as a source of important translational data. The SLN is the first place of meaningful interaction between the tumor and the patient’s immune system. As such, it may provide useful insights into the earliest aspects of anti-tumor immune response and the adverse effects on immunity induced by the tumor. The SLN immune microenvironment was initially examined by Cochran and colleagues with the examination of the immunologic features of the node in relation to other non-SLNs [41]. They developed the S100 immunostaining technique to identify small amounts of melanoma within SLNs [42]. This stain is quite sensitive and stains almost all melanomas. Interestingly, it also stains dendritic cells, the professional antigen presenting cells of the interfollicular region of the node. These early studies compared the area occupied by dendritic cells in SLN and compared it to the area in non-SLNs and found the area was significantly reduced in SLNs [43]. Moreover, the length of the dendritic processes extending from these cells (thought to be important for antigen uptake) were also shortened in SLNs. This finding was present regardless of the presence of tumor in the node, leading to the hypothesis that factors secreted from the primary tumor site were responsible for this immune modulation. Other changes that seem to precede the arrival of metastases in the SLN include increased lymphangiogenesis. There also appear to be differences in SLN that have metastases relative to those that do not. One intensive correlative analysis of SLNs found that those harboring metastases demonstrate more terminally differentiated lymphocytes and higher levels of immune checkpoint expression and regulatory T-cells relative to benign sentinel nodes, which show increases in dendritic cells, natural killer cell activation, and naïve T-cells [44]. Translational research continues now with the increasingly sophisticated tools of molecular biology and multiplexed spatial imaging.

Another observation derived from SLN biopsy was that the amount of radiotracer that was taken up and retained in SLNs was inversely related to the age of the patient [45]. This offers some insight into the clinical observation that, although older patients have a worse prognosis in melanoma, age is also inversely related to the probability of nodal metastasis. Indeed, Conway and colleagues reported that older age was highly significantly related to lower radioactivity counts in the node at the time of biopsy, which was independent of other variables. Ecker and colleagues later confirmed this finding in a much larger cohort and demonstrated multiple clinical correlates with these reduced counts on multivariable analysis, including worse melanoma-specific survival [46]. The biological basis of this appears to be, at least in part, through a decrease in HAPLN1, which occurs during aging. Loss of HAPLN1 is associated with increased endothelial permeability. Aging was associated with a decreased frequency of nodal metastases and an increased frequency of distant metastases in murine models. Restoring HAPLN1 in aged mice reconstituted endothelial integrity, and knocking it down in young mice made their lymphatic endothelium and metastasis pattern match that of aged mice. This new understanding may open new avenues of research in controlling metastasis in melanoma.

## 4. Conclusions

The lymphatic system appears to be the path of least resistance for the dissemination of melanoma, and lymphatic metastases have been recognized as important in the disease from the time of its earliest descriptions. Enormous effort has been devoted to understanding the process of lymphatic metastasis and how to best manage regional lymph nodes in patients with this disease. This work has yielded a current set of guidelines that allow for very accurate pathologic assessment of the status of regional lymph nodes that helps provide the most accurate prognostic estimates possible to guide therapy. The SLN procedure is a critical component of this program for many patients. The same procedure provides very good regional nodal disease control for patients, even for a majority of patients whose melanoma has spread to their nodes at the time of diagnosis. It achieves this through a minimally invasive process with very limited short- and long-term morbidity. While ongoing clinical progress, including in gene expression profiling and in neoadjuvant treatment of clinical melanoma nodal metastases, may alter the role of SLN biopsy in our treatment algorithms over time, it remains vital in many cases at this time.

## Data Availability

No new data were created or analyzed in this study.
